# Emergency Pancreatoduodenectomy: A Non-Trauma Center Case Series

**DOI:** 10.3390/jcm11102891

**Published:** 2022-05-20

**Authors:** Diana Schlanger, Călin Popa, Andra Ciocan, Cornelia Șofron, Nadim Al Hajjar

**Affiliations:** 1 Surgery Department, “Iuliu Haţieganu” University of Medicine and Pharmacy, Street Emil Isac no 13, 400023 Cluj-Napoca, Romania; schlanger.diana@yahoo.com (D.S.); andra.ciocan10@gmail.com (A.C.); cornelia.sofron@gmail.com (C.Ș.); na_hajjar@yahoo.com (N.A.H.); 2 Surgery Department, Regional Institute of Gastroenterology and Hepatology Prof. Dr. O. Fodor, Street Croitorilor no 19–21, 400162 Cluj-Napoca, Romania

**Keywords:** emergency pancreatoduodenectomy, emergency surgery, pancreatic surgery

## Abstract

(1) Background: Emergency pancreatoduodenectomy (EPD) is a rare procedure, especially in non-trauma centers. Pancreatoduodenectomy is a challenging intervention, that has even higher risks in emergency settings. However, EPD can be a life-saving procedure in selected cases. (2) Methods: Our study is a single-center prospective consecutive case series, on patients that underwent emergency pancreatoduodenectomies in our surgical department between January 2014 to May 2021. (3) Results: In the 7-year period, 4 cases were operated in emergency settings, out of the 615 patients who underwent PD (0.65%). All patients were male, with ages between 44 and 65. Uncontrollable bleeding was the indication for surgery in 3 cases, while a complex postoperative complication was the reason for surgery in one other case. In three cases, a classical Whipple procedure was performed, and only one case had a pylorus-preserving pancreatoduodenectomy. The in-hospital mortality rate was 25% and the morbidity rate was 50%; the two patients that registered complications also needed reinterventions. The patients who were discharged had a good long-term survival. (4) Conclusion: EPD is a challenging procedure, rare encountered in non-traumatic cases, that can be a life-saving intervention in well-selected cases, offering good long-term survival.

## 1. Introduction

Emergency pancreatoduodenectomy (EPD) is a rarely reported procedure, performed for complex pancreaticoduodenal injuries, with only a few cases being presented in medical literature. EPD has first been mentioned in trauma settings [1]. In non-traumatic indications, EPD has been reported sporadically, in a percentage of 0.3–3% [2]. Even though being recognized as a challenging and high-risk procedure, EPD still has an important role in some cases, when less invasive procedures fail to resolve the problem. Pancreatoduodenectomy (PD) is a technical complex procedure; the morbidity and mortality of the elective procedure has decreased in recent years especially due to a proper preoperative management, although morbidity rates remain high [3,4,5]. The lack of such preoperative preparation makes EPD even more demanding and challenging. The technical complexity of EPD is amplified by other underlying factors, such as malignancies, local infection, blood loss, coagulation disorders and a severely altered general condition [6]. As a non-trauma surgical center, we intend to present our experience with this type of intervention, in a short series of cases. Given the rarity of this procedure, we believe that every report of a significant case adds important scientific value.

## 2. Materials and Methods

Our study is a single-center prospective consecutive case series. The study has been conducted according to the PROCESS guidelines [7]. The participants were recruited from the patients operated in the Surgical Department of the Regional Institute of Gastroenterology and Hepatology Prof. Dr. O. Fodor Cluj-Napoca, a non-trauma digestive surgical center, between January 2014 and May 2021. The last follow-up information was in August 2021. The study was approved by the Ethical Committee of the Regional Institute of Gastroenterology and Hepatology Prof. Dr. O. Fodor Cluj-Napoca. The study protocol has been registered on clinicaltrials.gov, with the registration number NCT05139394.

The study included patients that underwent pancreatoduodenectomies in emergency settings. Since we are analyzing emergency surgical interventions, the pre-intervention optimization of the patient was minimal, with a short time interval for preparation for surgery. Regarding the intervention, all included patients underwent EPD with postoperative supportive treatment (including nutritional and hydro-electrolytic rebalancing treatment, antibiotics, and analgesics) conducted in the intensive care unit until stabilization and continued on the surgical ward. All surgeries were performed by the same operator (N.A.) and his team, with experience in hepatopancreatic surgery. Each patient was recommended to adhere to their follow-up consultations, in our hospital, every three months after discharge, for the first year and annually afterwards, for five years. Data was collected from the electronic system database. Each patient was assigned a numerical code, to assure anonymity and protection of personal data. We gathered different types of information, such as:

- Demographic data (age, sex, rural or urban area of living)
- Previous medical history (comorbidities)
- Reasons for emergency admission (symptoms and clinical signs)
- Preoperative investigations (blood work and imagistic investigations)
- Initial diagnosis
- Surgical intervention details (type of resection and reconstruction modality, blood loss, operative time)
- Definitive histopathological diagnosis of the resection specimen
- Postoperative investigations
- Patient evolution, including encountered complications
- Reinterventions
- Long term follow-up data
- Overall survival

## 3. Results

In the mentioned time interval, a total of 615 pancreatoduodenectomies were performed in our institution, only 4 cases being classified as emergency interventions, constituting a percentage of 0.65%.

Table 1 will present the demographic data, the indication for surgery, and the initial diagnosis of the four patients included in our case series.

Table 2 describes the details regarding the surgical intervention. All patients underwent surgical intervention less than 12 h after admission. The average operating time was 243.75 min, and the average blood loss was 425 mL.

In Table 3 we summarize the postoperative evolution of each case, including reinterventions, complications, intra-hospital deaths, in hospital stay and overall survival. The average in hospital stay was 18.75 days, while the average intensive care unit stay was 12.75 days.

### 3.1. Case 1

**Figure 1:**
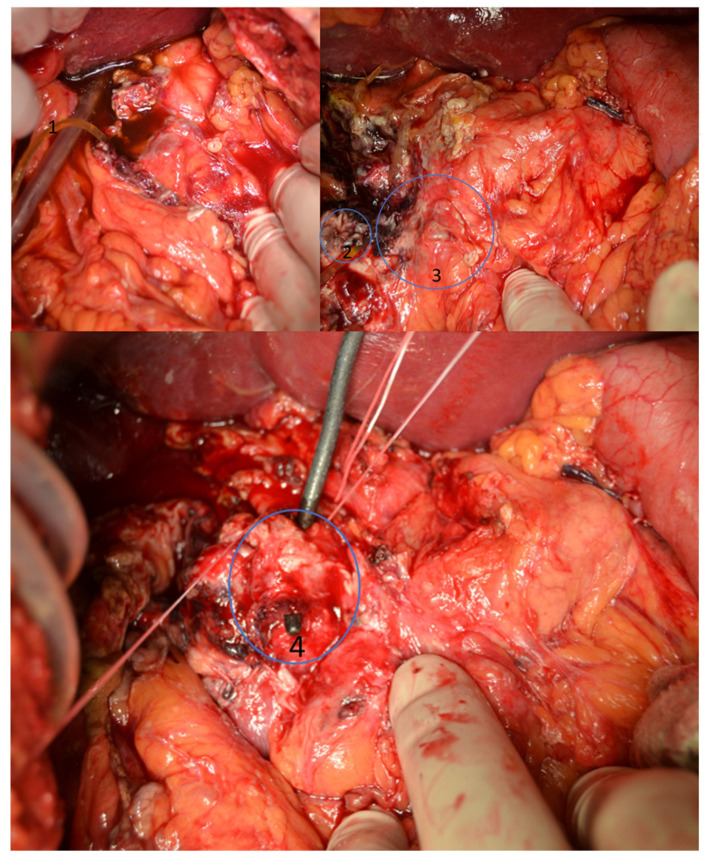
Intraoperative photo—the complex lesion of the duodenum, distal bile duct and pancreatic head. 1—Drainage tube placed in the bile collection, 2—Lesion of the duodenum, 3—Head of the pancreas, 4—Lesion of the distal common bile duct.

In the first case, the patient was transferred to our department from another hospital, after a complex iatrogenic lesion of the distal bile duct, ampulla, duodenum, and pancreatic head has occurred during a distal gastrectomy performed for perforated duodenal ulcer. At admission, the patient had a severely altered general state, which required immediate intervention. Emergency laparotomy identified a bile peritoneal collection (500 mL), the distal bile duct resected together with portions D1 and D2 of the duodenum and the gastric antrum; an external drainage tube was placed at the level of the common bile duct lesion (Figure 1).

A pancreatoduodenectomy was performed, with pancreaticogastric anastomosis, hepaticojejunal anastomosis and gastrojejunal anastomosis. The final histopathological diagnosis of the resection specimen showed acute necrotizing pancreatitis with acute duodenitis. The early postoperative evolution was eventless. No follow-up data is available since the patient did not attend the scheduled postoperative visits. The overall survival of the patient was almost 6 years.

### 3.2. Case 2

The second patient presented in emergency settings with melena, with a hemoglobin level of 7.9 g/dL at presentation. An emergency gastroscopy was carried out: large quantities of blood were found in the duodenum, without the identification of an exact source. The CT scan showed a tumor at the level of the D2 portion of the duodenum, measuring 62/66/76 mm, in proximity with the inferior vena cava and the right kidney. The condition of the patient altered quickly, with hemodynamic instability, therefore emergency laparotomy has been decided. A classical Whipple PD was performed with pancreaticogastric anastomosis, hepaticojejunal anastomosis and gastrojejunal anastomosis. The final histopathological diagnosis showed a gastrointestinal stromal tumor, stage T3N0M0, without lympho-vascular invasion, with free resection margins (R0), and moderate grade differentiation (G2). Ten days after the initial surgery, an intestinal obstruction occurred, by stenosis at the level of the jejunostomy: reintervention has been prompted and a segmental enterectomy with anastomosis was performed. The patient’s evolution was marked by a superficial wound site infection, a Clostridium Difficile enterocolitis, an internal jugular vein thrombosis (treated with anticoagulants) and an omental bursa fluid collection (managed conservatively, with complete resolution at 1 month after surgery). The patient had a close surgical and oncological follow-up. Two years after surgery, peritoneal metastases were suspected, but were infirmed through biopsy. Three years after the EPD, the patient is reoperated for intestinal obstruction and bile duct stenosis: adherence dissection and redo of the hepaticojejunal anastomosis was performed. In the present, almost 5 years after the EPD, the patient is alive and continues the treatment and follow-up as prescribed by the oncologist.

### 3.3. Case 3

**Figure 2:**
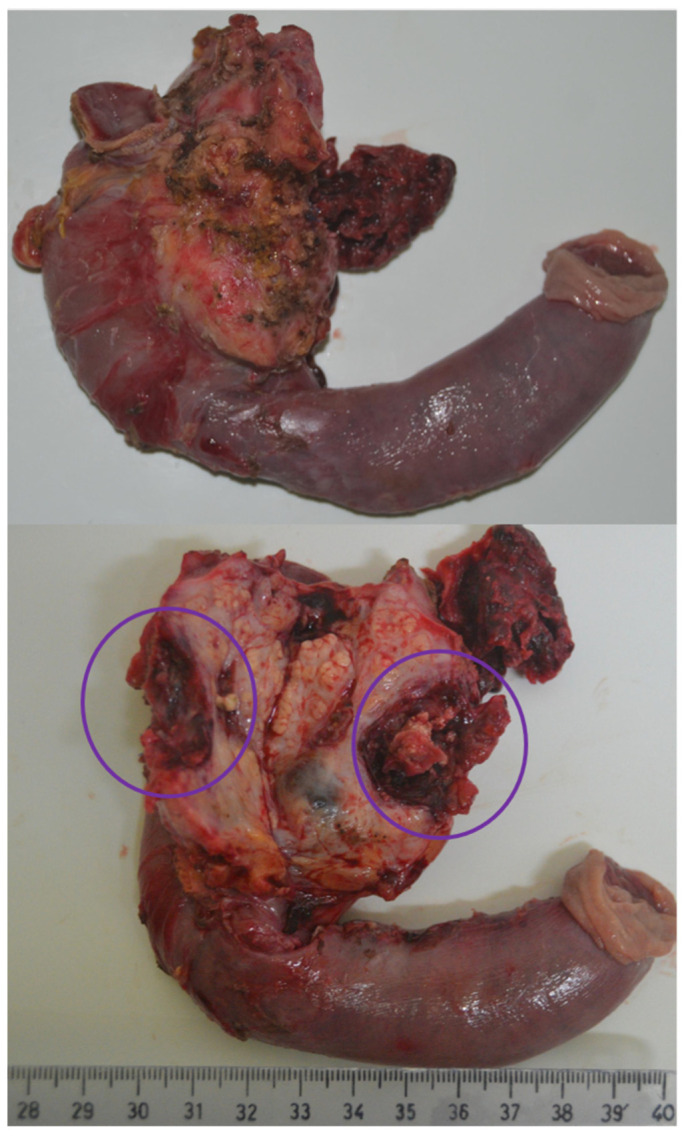
Resection specimen, with emphasis on the pseudoaneurysm of the pancreaticoduodenal artery.

The third patient had a medical history of chronic ethanolic pancreatitis with numerous episodes of acute pancreatitis. He presented in our emergency unit with melena; the hemoglobin level was 8.7 g/dL at presentation. A computer-tomography was carried out, which described chronic pancreatitis and a pseudoaneurysm of the pancreaticoduodenal artery localized near the uncinate process. The general state of the patient was rapidly aggravating, with hemodynamic instability, hemoglobin drop, and initiation of vasoactive support. Due to the patient’s condition, emergency surgery was performed: pylorus-preserving pancreatoduodenectomy with pancreaticojejunostomy, hepaticojejunostomy and duodenojejunostomy. (Figure 2) The postoperative evolution was uneventful; the routine follow-up visits at 3 and 6 months showed favorable evolution. In the present, the patient is alive, at about 4 years after the EPD.

### 3.4. Case 4

The fourth patient presented in our hospital with severe abdominal pain and profoundly altered general state. A CT scan was performed, describing encapsulated pancreatic collections after an acute pancreatitis episode and a ruptured pseudoaneurysm of the gastroduodenal artery with active bleeding; thrombosis in the inferior vena cava and a thrombus in the right atrium were also described. The blood tests showed a hemoglobin level of 10.2 g/dL, amylase of 148 U/L and lipase of 484 U/L. Since the condition of the patient altered quickly, emergency laparotomy was decided by a multidisciplinary commission. EPD was performed with reconstruction by pancreaticojejunal, hepaticojejunal and gastrojejunal anastomoses. After the EPD, a cardiovascular surgery consultation recommended surgical intervention to remove the thrombus from the right atrium: the patient refuses the intervention. The evolution of this case was unfavorable with hepatic ischemia and advanced thrombosis of the inferior vena cava. A surgical complication also occurred with hepaticojejunal anastomosis fistula—reintervention with reconstruction of this anastomosis was performed. Overall, the evolution was unfavorable, with the development of multiple organ dysfunction syndrome; the death of the patient occurred at 20 days after the pancreatoduodenectomy.

## 4. Discussion

### 4.1. Emergency Pancreatoduodenectomy as a Rare Surgical Procedure in Non-Traumatic Cases

The presented cases reflect the limited experience of our center with EPD. In both our experience, and reported medical literature, this is an exceptional procedure, carried out in less than 3% of cases, even in high-experience pancreatic surgery centers [2]. There have been very few case series reported on this subject, most articles being isolated case reports. For example, Lissidini et al. [8] reported 5 cases in a 9-year interval (3%), Strobel et al. [9] reported 10 cases in a 11-year time period, Nentwich et al. [10] reported 10 cases in 9 years (1.7%), Z’graggen et al. [11] reported 4 cases in 7 years interval (0.96%), Standop et al. [6] reported 6 cases in 19 years (2%), Gulla et al. [12] reported 3 cases in 11 years (0.3%), while Tsai et al. [2] reported 6 cases in 14 years (0.6%). As can be observed, the report of our 4 cases in a 7-year period aligns with the current medical information, underlining that EPD is an uncommon and last resort intervention used only when less invasive procedures fail. There is one other published study [13] with a larger patient sample, but the definition of the PD that was classified as emergency was different, based on several parameters but not considering the delay from admission to surgery.

Complex duodenopancreatic traumatic injuries are uncommon but severe cases might need EPD. Although there are more cases of trauma EPD presented in medical literature, this remains a rare intervention with high associated risks. However, we need to define a clear difference between EPD in traumatic and non-traumatic injuries: the same surgical intervention gains totally different perspectives based on its underlying pathophysiology. EPD in trauma cases has similar results, being considered an intervention with high mortality rate (around 40%), but with good overall results and minimal long-term complications in well selected patients [14,15,16,17,18].

### 4.2. Indication for Surgery

In our case series, in 3 patients, acute hemorrhage (upper gastrointestinal bleeding in 2 cases and intraperitoneal hemorrhage in 1 case) constituted the indication for surgery and in 1 patient, a postoperative iatrogenic lesion led to EPD.

A good selection of the patients is essential for assuring the best outcome. The high risks associated with EPD need to be put in balance with the potential benefits that this intervention might assure. We need to be critical with the evaluation of the indication for surgery in the presented cases. Although we can identify well established reasons for choosing an EPD in all patients, we need to address the last case, with unfavorable evolution, that presented major associated cardiovascular conditions. Even though the proper management of this patient was decided by a multidisciplinary team, we can now retrospectively discuss whether an EPD was appropriate since the patient refused definitive cardiovascular treatment, this decision ultimately leading to his death. We need to flag the problems associated with this case for reference for future decisions of this sort.

Between the published cases in medical literature, uncontrollable bleeding seems to be the most common indication for EPD. Iatrogenic injuries are another frequent indication for EPD [2,8,11,19,20]; in our case, a postoperative complication led to this intervention, but there have been reports of lesions after esophagogastroduodenoscopy or endoscopic retrograde cholangiopancreatography that led to EPD. In addition to the aforementioned indications, there have been reported other causes that needed EPD, such as digestive tract perforations. The second presented case was a duodenal bleeding GIST; four other reported cases of EPD had the same etiology [9,21,22,23]. We have also reported two cases of bleeding pseudoaneurysms (pancreaticoduodenal artery and gastroduodenal artery); only one case of EPD for bleeding pseudoaneurysm of the pancreaticoduodenal artery was reported. [24,25]

Even though interventional radiology methods need to be taken into consideration in this type of cases, the evolution of our reported cases did not permit the use of these techniques: the massive bleeding caused hemodynamic instability and definitive surgical control was needed. Especially in bleeding pseudoaneurysms, such as our presented cases, an embolization through an interventional radiology technique can either be a definitive treatment or at least a temporary treatment for assuring a proper preoperative preparation of the patient. Since EPD is associated with important risks, and uncontrollable bleeding is one of the most common indications for this surgery, a closer attention should be directed on interventional radiology methods capable to solve some of these problems. The management of the acute duodenopancreatic complex bleeding should be carried out through a combination of interventional radiology and surgery, surgical intervention being reserved for cases in which embolization has failed or in unstable patients. [26,27]

### 4.3. Surgical Intervention

Classic Whipple EPD was performed in 3 cases and a pylorus preserving EPD was performed in another case; immediate reconstruction was carried out in all cases. Staged reconstruction has been reported in some cases [10,28,29]; we preferred the one-stage reconstruction just as in elective settings. The reconstruction of the pancreatic remnant was carried out by a pancreaticogastric anastomosis in 2 cases and by a pancreaticojejunal anastomosis in the other 2 cases. The pancreatic reconstruction has registered a high variability in surgical technique in elective surgery, and especially in EPD: anastomosis between the pancreas and the digestive tract (stomach or jejunum) [6,19,23,30,31], closure of the pancreatic stump [8] or pancreaticostomy [22,32].

The operative time and intraoperative blood loss vary significantly. We registered operative times between 190 and 300 min, such as the ones reported in elective surgery (200–400 min) [3]. In the reported cases of EPD, even higher operative times were reported, around 500 min, depending on the complexity of the procedure. [9,10] Regarding blood loss, an average of 400 mL is reported in elective PD [3]; we reported between 300 and 700 mL, with an average of 425 mL between the four cases.

### 4.4. Early Postoperative Evolution

In half of the cases, the postoperative evolution was uneventful (50% morbidity rate). The other two cases have encountered various complications and required reintervention as well. The morbidity rate in EPD is reported between 80% and 90% [6,8,12]. The most common complication registered in EPD is pancreatic fistula; we have not encountered it in our case series. One reintervention was carried out for intestinal obstruction and the other for a biliary fistula.

We had one in hospital death, a mortality of 25% being registered. A mortality rate between 17% and 40% is reported in EPD [2,6,8,10,11,24]. The fourth case had complex cardiovascular associated problems, and unfortunately, refused any cardiac surgery; the unfavorable evolution of this patient underlines once again the importance of the selection of the patients who might benefit from such an extensive intervention.

A comparison with elective PD results shows higher in-hospital mortality and reintervention rates for EPD. Nowadays, the reported in-hospital mortality rate in elective setting is less than 5% in medical literature [4], with similar results in our institution (5.9%), especially due to effective preoperative management. The reintervention rate in elective surgery is around 10% [4] both in medical literature and our center. However, morbidity rates remain important even in elective settings (around 60%) [33], our EPD cases showing similar results. We can therefore observe that a downside of EPD remains the high mortality rate due to the lack of preoperative planning, which is impossible to compensate by only postoperative support.

### 4.5. Long-Term Evolution

Between the three cases that were discharged, in one case no follow-up data were available, one had a more complicated evolution, with more reinterventions, while one case had a favorable long-term evolution. The second case, which registered the most complex evolution, was the only oncological patient of this case series, with multiple complications and reinterventions. However, the overall survival of the first three cases is good, two patients being alive at the time of this study, while the other patient had a postoperative survival of about 6 years. Information regarding the follow-up of patients and overall survival is scarce in medical literature [8,21,23,29,32,34,35,36,37,38].

The long-term survival after PD in elective setting is mainly dictated by the underlying histopathology of the resected specimen—since the most common indication for PD is pancreatic adenocarcinoma, long term prognosis is usually poor (an average of 22 months for overall survival in our center). Therefore, a better long-term survival is registered in EPD, which can have benign or indolent pathologies as an underlying indication for surgery. This once again reinforces the fact that EPD is a life-saving procedure that has major benefits especially when a good selection of the candidates is carried out.

### 4.6. Strengths

Our study is one of the few case series that reports the use of EPD. Since EPD is a rare procedure, we believe that every report is important to further determine the correct management in these cases. The prospective design of the study helps capture all the important details of the patients.

### 4.7. Limitations

The small sample size is one of the biggest limitations of this report, but the rarity of the procedure needs to be taken into consideration when analyzing this factor.

## 5. Conclusions

The four presented cases underline the utility of EPD in well-selected non-traumatic cases. Even though the lack of preoperative preparation of the patient increases the risks of this procedure, favorable outcomes can be reached and EPD can be a life-saving procedure.

## Figures and Tables

**Table 1:** Demographic details, motive of presentation and diagnosis.

No	Year	Age (Years)	Sex	Living Area	Reason for Emergency Presentation	Diagnosis
1	2014	65	M	R	Postoperative complication	Complex iatrogenic lesion of the distal bile duct, duodenum, and head of pancreas
2	2016	47	M	U	UGIB-melena	Bleeding duodenal tumor
3	2017	44	M	U	UGIB-melena	Fistulized pancreaticoduodenal artery aneurysm in the duodenum
4	2021	53	M	R	Upper abdominal pain. Altered general state.	Ruptured gastroduodenal artery aneurysm

M—male, R—rural, U—urban, UGIB—upper gastro-intestinal bleeding.

**Table 2:** Details regarding the surgical intervention.

No	PD Type	Reconstruction of Pancreatic Remnant	Operative Time (min)	Blood Loss (mL)
1	PD CW	PG	190	300
2	PD CW	PG	300	300
3	PD PP	PJ	210	700
4	PD CW	PJ	275	400

PD—pancreatoduodenectomy, CW—classic Whipple, PP—pylorus-preserving, PG—pancreaticogastric anastomosis, PJ—pancreaticojejunal anastomosis.

**Table 3:** Data on the postoperative evolution of the patients.

No	In Hospital Stay Days	Intensive Care Unit Stay Days	Complication	Reinterventions	Intra Hospital Death	Postoperative Survival Time (Days)
1	12	7	-		-	2047
2	31	19	Intestinal obstruction Wound infection Internal jugular vein thrombosis Clostridium Difficile infection	Yes—Segmental enterectomy	-	1698 (alive)
3	12	5	-		-	1548 (alive)
4	20	20	HJ anastomotic fistula Thrombosis of IVC and iliac veins Left hepatic lobe ischaemia	Yes—Redo of the HJ	Yes	20

HJ—hepaticojejunostomy, IVC—inferior vena cava.

## Data Availability

The datasets used and/or analyzed during the current study are available from the corresponding author on reasonable request.
